# Cardiac inotropic rebound effect after washout of acetylcholine is associated with electrophysiological heterogeneity in Langendorff-perfused rabbit heart

**DOI:** 10.3892/etm.2014.1486

**Published:** 2014-01-15

**Authors:** HAIJIAN LUO, JUNQIANG SI, FENGJIE ZHANG, ZHENYU YANG, RUXING WANG

**Affiliations:** 1 Department of Physiology, Shihezi University School of Medicine, Shihezi, Xinjiang 832003, P.R. China; 2 Department of Cardiology, Wuxi People’s Hospital Affiliated to Nanjing Medical University, Wuxi, Jiangsu 214023, P.R. China; 3 Institute of Cardiovascular Sciences, The University of Manchester, Manchester M13 9PL, UK

**Keywords:** acetylcholine, vagus nerve, triggered activity, heterogeneity, rabbit heart

## Abstract

Cardiac electrophysiological heterogeneity related to the washout of acetylcholine (ACh) remains incompletely characterized. The aim of this study was to examine whether positive cardiac inotropic action is associated with electrophysiological heterogeneity between the atrium and the ventricle after ACh perfusion and washout. Epicardial monophasic action potentials (MAPs) from the right ventricle and right atrium, as well as cardiac contractility, were recorded from isolated Langendorff-perfused rabbit hearts using MAP electrodes and a force transducer. The results indicated that rebound positive inotropic actions were induced by ACh washout with adrenaline preconditioning. This effect was accompanied by an increase in MAP amplitude (MAPA) in the right ventricle but not the right atrium. These findings indicate that cholinergic muscarinic stimulation may lead to positive cardiac inotropic action followed by changes in regional electrophysiological heterogeneity between the atrial and ventricular myocardium. Therefore, we hypothesize that electrophysiological heterogeneity is an underlying cause of arrhythmogenesis as well as hemodynamic disturbance elicited by sudden termination of vagus stimulation.

## Introduction

Cardiac responses to the continuous infusion of acetylcholine (ACh) or direct vagal nerve stimulation at a constant intensity diminish over time, a phenomenon known as desensitization (1–4). However, rapid washout of ACh or sudden withdrawal of muscarinic cholinergic stimulation has a positive effect that is opposite to the inhibitory actions of ACh, a phenomenon known as rebound, which may induce cardiac hemodynamic disturbances and arrhythmias (5–7).

The existence of complex regional differences in the electro-mechanical properties of the healthy adult heart has been confirmed (8–10). Several published studies describe the role of cardiac electrical heterogeneity in the re-entry and triggered activities that are responsible for atrial fibrillation, and in ventricular tachycardia and inherited ion channelopathies. However, the impact of ACh washout on regional electrophysiological heterogeneity in the isolated perfused heart remains unclear (9–11).

The present study was conducted to determine whether the washout of ACh is associated with regional electrophysiological heterogeneity accompanied by positive inotropic rebound action. As ACh washout mimics the sudden withdrawal of vagus stimulation, a factor that may trigger arrhythmia, electrophysiological characterization of ACh-induced inotropic effects may improve the understanding of arrhythmogenesis.

## Materials and methods

### Langendorff perfusion

All experiments were performed according to ethical guidelines approved by the Shihezi University Institutional Animal Care and Use Committee. Isolated Langendorff-perfused rabbit hearts were prepared as previously described (12,13). Briefly, male New Zealand white rabbits (n=7; mean weight, 2.6±0.3 kg) were anaesthetized with sodium pentobarbital (70 mg/kg i.v.) delivered with heparin (1000 U/kg i.v.). The hearts were excised, aortae were cannulated rapidly, and the hearts were perfused with modified Krebs-Henseleit (K-H) solution (composition in mM: 118 NaCl, 4.5 KCl, 11.0 glucose, 24.9 NaHCO_3_, 1.1 MgSO_4_, 2.5 CaCl_2_ and 1.1 KH_2_PO_4_). The temperature was held at 37°C and the pH was maintained at 7.4 by continuously bubbling with a mixture of 95% O_2_ and 5% CO_2_. For experiments performed in the Langendorff mode, the heart was perfused retrogradely with a modified K-H solution in a constant perfusion pressure mode (75 cm H_2_O).

### Monophasic action potential (MAP) recording

Two Ag-AgCl pressure-contact MAP electrodes were positioned in the apical epicardial region of the right atrium (RA) and right ventricle (RV). MAP data and cardiac contraction curves were recorded using an amplifier, an A/D-converter, and a computer with the BL-420E Biological Experimental System (Taimeng Technology Co., Ltd., Chengdu, China) (13,14).

### Drug infusion

Isolated rabbit hearts were subjected to retrograde aortic perfusion of ACh solution (10^−5^ mol/l) followed by rapid washing out with modified K-H solution. Subsequently, adrenaline (10^−4^ mmol/l) was infused to force recovery of contraction to baseline. ACh perfusion followed by rapid washout was repeated when the contraction curve recovered to baseline. For all drugs, the infusion velocity was 0.05 ml/sec.

### Statistical analysis

Only stable MAPs prior to drug infusion with constant amplitude (>4.5 mV) were accepted for analysis (n=7). MAP amplitude (MAPA) was defined as the distance from the diastolic baseline to the crest of the MAP plateau phase. The duration of MAP was measured manually at the time of 90% repolarization (MAPD_90_) and the mean value was calculated from three consecutive signals. MAPA, and the inotropic amplitude, onset and duration time are presented as the means ± standard deviation (SD). Results were analyzed using the Student’s t-test with P<0.05 denoting statistically significant differences.

## Results

### ACh-induced positive inotropic action

**Figure 1:**
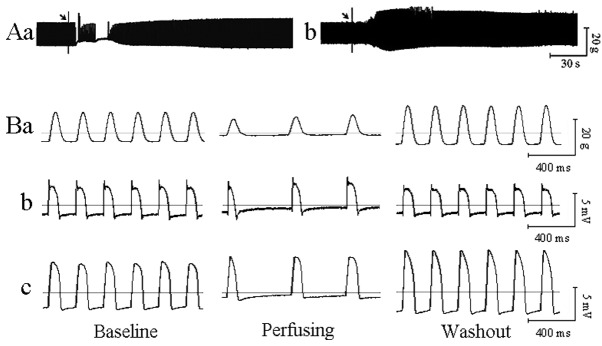
Positive inotropic action and MAP curves induced by ACh. (Aa and b) and (Ba) show the changes in myocardial contractility prior to and following ACh washout. The arrowheads in (A) denote the initiation of ACh perfusion and rapid washout. (Bb and Bc) show ACh-induced changes on the MAPs of the right atrium and right ventricle, respectively.

Maximum rebound effects were observed between 100 and 460 sec and recovery to baseline was observed following the infusion of ACh (760±51 sec). The maximum rebound rate was 78.53±27.86% (Fig. 1Aa and b and Bc).

### Changes in MAPs following ACh washout

Washout of ACh induced higher MAPA in the RV (Fig. 1Bc) but not in the RA (Fig. 1Bb). This effect was accompanied by positive inotropic action (Fig. 1Ba) and changes in MAPs. Only the change of MAPA in RV was statistically significant after the washout of ACh (Table I).

## Discussion

Previous studies have demonstrated that the ACh-induced enhancement of myocardial contractility is related to the following mechanisms, the majority of which act at the cell and tissue level: i) following ACh washout, inhibition of adenylate cyclase is abolished, which increases the sensitivity of the myocardial cells to adrenaline (15,16); ii) as ACh increases the levels of nitric oxide and cGMP, resulting in the activation of phosphodiesterase II, which downregulates intracellular cAMP levels, a phenomenon involving the rebound of the cAMP level and an increase in the number of opened Ca^2+^-channels occurs after the elution of ACh (13,17,18); iii) ACh activates Ca^2+^-channels in myocardial cells via the muscarinic M1-receptor (19,20); iv) ACh acts on M2-receptors to facilitate Ca^2+^ influx via Na^+^/Ca^2+^ exchangers, but fails to affect the contractility of the rabbit heart (21,22); v) ACh activates adenylate cyclase via the G-protein βγ-subunit to increase intracellular cAMP levels (13,15) and vi) the sensitivity of myocardial myofibrils to Ca^2+^ increases following ACh washout (23).

Complex regional differences in the electro-mechanical properties of the healthy adult heart, particularly in the ventricle, have been confirmed. Electro-mechanical features are affected by different pathological conditions, including myocardial ischaemia and cardiac hypertrophy (8). In the present study, ACh washout following adrenaline pretreatment resulted in a higher MAPA accompanied by positive inotropic action in the RV (Fig. 1Bb) but not in the RA (Fig. 1Ba), as assessed by the MAP technique.

ACh-induced positive inotropic actions have been documented in previous studies by using individual myocardial cells or muscle strips, ventricular muscle and other isolated tissue blocks; however, it is necessary to perform integrated confirmative studies at both the organ and body level. The atrium and ventricle, as two functional components, may electrically and mechanically differ. Consequently, integrated and *in vivo* research methods should be the basic means of investigating these actions. In the present study, regional electrophysiological heterogeneity was exhibited as the rebound positive inotropic action induced by the washout of ACh, which may be a factor leading to cardiac arrhythmia (24,25).

## Figures and Tables

**Table I:** Effects of ACh (10^−5^ mol/l) on epicardial MAPs in isolated perfused rabbit hearts (n=7).

Variable	Baseline	Perfusion	Washout
RA			
MAPA (mV)	6.3±1.5	5.0±1.4^a^	5.8±1.9
MAPD_90_ (ms)	128.4±17.6	89.6±16.8	135.6±12.5
RV			
MAPA (mV)	8.5±2.8	6.1±2.3^a^	10.6±3.7^a^
MAPD_90_ (ms)	139.8±13.5	92.7±12.5^a^	143.2±15.6

Results are expressed as the means ± SD. RA, right atrium; RV, right ventricle; MAPA, monophasic action potential amplitude; MAPD_90_, monophasic action potential duration at 90% repolarization.

a P<0.05 vs. baseline.
